# A new possible marker: can pennation angle defined by ultrasound predict the frailty?

**DOI:** 10.1007/s40520-023-02663-w

**Published:** 2024-03-05

**Authors:** Busra Yurumez, Yavuz Metin, Volkan Atmis, Mursel Karadavut, Sinan Ari, Emine Gemci, Seher Yigit, Funda Seher Ozalp Ates, Melih Gaffar Gozukara, Ceren Kaplankiran, Caglar Cosarderelioglu, Ahmet Yalcin, Sevgi Aras, Murat Varli

**Affiliations:** 1 Division of Geriatrics, Department of Internal Medicine, Ankara University Faculty of Medicine, Ibn-I Sina Hospital, Altindag, Ankara, Turkey; 2 Department of Radiology, Ankara University Faculty of Medicine, Ankara, Turkey; 3 Department of Biostatistics and Medical Informatics, Manisa Celal Bayar University Faculty of Medicine, Manisa, Turkey; 4 Ankara Sincan Public Health Center, Ankara, Turkey

**Keywords:** Rectus femoris, Frail, Muscle, Pennation angle, Thickness

## Abstract

**Background:**

Frailty indicates older people who are vulnerable to stressors. The relation between ultrasonographic parameters of muscle and frailty among older people has yet to be investigated.

**Aims:**

The aim of the study is to investigate the relationship between frailty and the ultrasonographic measurements of the rectus femoris muscle (RFM).

**Methods:**

This cross-sectional study included 301 participants who were ≥65 years. The FRAIL questionnaire assessed frailty. The thickness, cross-sectional area (CSA), fascicle length, pennation angle (PA), stiffness, and echogenicity of RFM were assessed by ultrasound. The accuracy of parameters in predicting the frailty was evaluated by ROC analysis.

**Results:**

Of all 301 participants, 24.6% were frail. Pre-frail and frail participants had significantly lower thickness (*p* = 0.002), CSA (*p* = 0.009), and fascicle length (*p* = 0.043) of RFM compared to robust. PA was significantly lowest in frails (*p* < 0.001). The multivariate logistic regression analysis showed that PA values lower than 10.65 degrees were an independent predictor of frailty (OR = 0.83, 95% Cl: 0.70–0.97, *p* = 0.019). Results of ROC analysis demonstrated a satisfactory result between the PA and frailty (AUC = 0.692, *p* < 0.001).

**Discussion:**

Thickness, CSA, and PA of RFM were found to be lower in frail subjects, which may indicate the changes in muscle structure in frailty. Among all parameters, lower PA values were independent predictors of frailty. These findings may indicate a novel ultrasound-based method in frailty, that is more objective and unrelated to the cross-sectional evaluation.

**Conclusions:**

Ultrasonographic measurements of RFM, especially the lower PA may predict frailty in older people. As an objective and quantitative method, PA may be used to define frailty with acceptable sensitivity.

## Introduction

All over the world, the number and proportion of older adults are rapidly increasing, and age-related diseases are becoming a challenge for aging populations over the years [1, 2]. Frailty prevalence increases with age and is associated with an increased risk of negative clinical outcomes such as dependency or death in response to acute stressors [3]. Different definitions were developed to conceptualize frailty. These definitions have different aspects, but they also have similar determinants such as weight loss, muscle weakness, and limitation in mobility [4]. Frailty is shown to be closely related to muscle mass, quality, and function. Different frailty definitions focusing on different aspects of the syndrome may be complex, time-consuming, and depending on the patient’s performance in the clinical practice. For example, Fried frailty phenotype, a reliable and common method to evaluate frailty, includes measuring hand-grip strength, walking speed or evaluating the recent physical activity that may be affected by acute problems or cross-sectional performances of people. Also, the frailty index is based on a variety of age‐related deficits and takes longer time to complete [4, 5]. There is an unmet need for a simple, objective, and practical method to diagnose frailty.

Recently, ultrasound has emerged as an alternative method to assess muscle mass and quality. Ultrasound, a simple, practical, non-invasive, and cost-effective method, is also important for not being related to patient cooperation, cognitive status, or volume status [6, 7]. Muscle thickness, cross-sectional area (CSA), fascicle length, stiffness, and pennation angle (PA) of lower limb skeletal muscles were evaluated in different studies, to estimate the skeletal muscle mass in sarcopenia [8–10]. Rectus femoris muscle (RFM), a part of the quadriceps muscle group, is frequently evaluated in the literature to estimate skeletal muscle mass with high spatial ultrasound resolution and the highest intra- and inter-rater reliability [10–12]. The relation between ultrasonographic parameters of skeletal muscle and frailty among older people was scarcely investigated [13, 14]. Shan et al. reported that the thickness and quality of skeletal muscles of the lower extremities were affected by the frailty status [13]. In an early study, echo-intensity detected by ultrasound was shown to be increased in frail patients compared to robust ones. The same study reported lower muscle thickness in frail subjects [15].

As muscle mass and quality are important in assessing frailty, the current study hypothesis is that in frail older adults, the structural characteristics of the muscles may be disrupted. In this context, our aim is to investigate the relationship between the frailty and the parameters obtained by ultrasound of the RFM: thickness, CSA, fascicle length, stiffness, PA, and echogenicity. Assessing the muscle characteristics by a non-invasive and easily applicable method may help to diagnose frail older adults in clinical practice.

## Methods

### Study design and participants

In the current cross-sectional study, we recruited 301 participants aged 65 years and older and admitted to outpatient clinics of a tertiary health center, from March 2021 to December 2021. Sociodemographic data, comorbidities, and anthropometric measurements of all participants were recorded, and a comprehensive geriatric assessment was applied. Ultrasonographic assessment of RFM was performed by the same radiologist. Participants with severe dementia, acute illness, end-stage organ failure, paraplegia/hemiplegia, and subjects who were immobile, unable to understand the study protocol, or hospitalized in the last 30 days were excluded. All participants provided written informed consent before they participated in the study. The study protocol was approved by the local research ethics committee of Ankara University (number: I6-392-21) and conducted in accordance with the Declaration of Helsinki.

### Comprehensive geriatric assessment

Two trained internal medicine specialists on the geriatric fellowship program interviewed all participants and sociodemographic data of the participants such as age, gender, marital status, education level, concomitant chronic diseases, and routine laboratory measurements were recorded. Following that, basic functional abilities, and level of dependence in the daily activities of participants were assessed by Katz Index of Independence in Activities of Daily Living Scale (Katz ADL) which consists of six items. Each functional ability is scored on a Likert scale of 0–3 according to the dependence level and the total score is calculated out of 18 [16]. Lawton Instrumental Activities of Daily Living Scale (Lawton IADL) assessing higher functional abilities, is rated dichotomously, “0” for dependence and “1” for independence [17]. Lower scores indicate a higher level of dependency in activities for both scales. To assess the nutritional status mini-nutritional assessment-short form (MNA-SF) was used. According to MNA-SF: a score of 7 and lower indicated “malnutrition”, scores between 8 and 11 indicated the “risk for malnutrition”, and the scores ≥ 12 represented the “normal nutritional status” [18]. The physical activities of participants were evaluated by the Turkish form of the International Physical Activity Questionnaire-short form, which has 7 items questioning vigorous-intensity physical activity, moderate-intensity physical activity, walking, and sitting [19].

Anthropometric parameters including height, weight, and calf circumference (CC) were measured. BMI was calculated using the following formula: weight (kg)/height^2^ (m^2^). Participants with BMI over 30 kg/m^2^ were evaluated as obese. CC was measured from the largest circumference of the calf without compressing the subcutaneous tissue, by a flexible measuring tape. For optimization of measurement, the patients were placed in a supine position with their left knee raised and a 90-degree angle was created between the calf and thigh. CC is showed to be correlated with muscle mass [12] and values lower than 31 cm were considered as low muscle mass [20]. Muscle strength was assessed by hand-grip strength which was measured by a Takei hand-grip dynamometer (Takei Scientific Instruments, Niigata, Japan) and low muscle strength was defined as <27 kg for men, <16 kg for women. The participants were asked to grasp the dynamometer between the fingers and the palm and squeeze as hard as they can, in a sitting position with the elbow at 90″ flexion. This procedure was repeated three times and the maximal values for each participant were recorded [21]. Physical performance was assessed by usual gait speed. Participants who walked slower than 0.8 m/s were assessed as having low physical performance [22].

### Assessment of frailty status

Frailty was assessed by the FRAIL questionnaire, which is a practical and valid instrument for screening frailty [23]. The FRAIL questionnaire consists of 5 dichotomous items, that question fatigue, resistance, ambulation, concomitant illnesses, and weight loss. One point was given for each “yes” answer and total scores range from 0 to 5. Participants were categorized into 3 groups according to the total score: robust (0 points), pre-frail (1–2 points), and frail (3 or more points) [24].

### Ultrasonographic assessment of rectus femoris muscle

Ultrasound images of the RFM were obtained by a B-mode two-dimensional ultrasound (LOGIQ E9, GE Healthcare, Wisconsin, USA with a linear transducer (3.1–10-MHz probe; 9L). All the ultrasonographic assessments were performed by the same sonographer, with at least 10 years of experience. The sonographer was blinded to the frailty status of the participants. During the sonographic assessment, participants were in a supine position with legs at rest, knees extended, and muscles relaxed. As it was important to avoid compression of the muscle tissue, plenty of contact gel was applied during the investigation. The linear probe was placed perpendicular to the long axis of the thigh and scans were acquired from the mid-point of the distance between the greater trochanter and the lateral condyle of the femur. Muscle thickness was defined as the perpendicular distance between the deep and superficial aponeuroses of the rectus femoris muscle and reported in millimeters (mm). CSA of the rectus femoris was measured from the same point in the transverse plane and expressed in millimeter square (mm^2^). Borders of the muscle were detected, and CSA was calculated manually from the images. Fascicle length is defined as the length of the fascicular path between the insertions of the fascicle into the superficial and deep aponeurosis. The angle between the muscle fascicle and the echo of deep aponeurosis is defined as PA in the longitudinal ultrasound images. Muscle echogenicity was considered normal if it was similar to the echogenicity of the subcutaneous tissue. Whereas it was defined as hyperechoic if muscle echogenicity was higher than subcutaneous tissue. All quantitative measurements were repeated three times and the mean values were recorded. Muscle stiffness (kilopascal, kPa) was measured using shear wave elastography (SWE). These measurements were done using a linear 3.1–10-MHz probe (9L, GE Healthcare). Six measurements were made from the central part of each muscle, and the mean value was recorded. Measurements were made using a circular ROI with an area of 20 mm^2^.

### Statistical analysis

All statistical analyses were performed using Statistical Package for the Social Sciences (SPSS) for Windows 11.5 (SPSS Inc., Chicago, IL, USA). To determine if there is a difference between the groups (robust, pre-frail, and frail) in terms of ultrasonographic parameters, the sample size was calculated by considering an effect size of 0.25 (medium), a power of 0.90, and an alpha of 0.05. It was determined that at least 69 people in each group should be included in the study. Sample size calculation was made with G*Power 3.1.9.7 program. The Kolmogorov–Smirnov test was used to assess the assumption of normality. Normally distributed continuous variables were expressed as mean ± standard deviation while the continuous variables that do not have normal distribution were expressed as median (minimum–maximum). Differences between the three groups (robust, pre-frail, and frail) were tested using the One-Way ANOVA/Kruskal–Wallis Test. Relationships between ultrasonographic measurements of RFM and FRAIL score were determined by the Spearman Correlation Coefficient. Univariate logistic regression analysis was used to analyze the factors leading to the risk of frailty. A two-sided *p* value <0.05 was considered as statistically significant. A multivariable logistic regression model was used to predict potential risk factors of the risk of frailty. The variables which had a significance level of *p* < 0.20 from the univariate analysis were identified as candidate variables for the multivariable model. The multivariable logistic regression model was created with Backward LR method.

The accuracy of the test was measured by the area under the ROC curve (AUC), an area close to 1 represented a better prediction ability. The sensitivity, specificity, positive and negative predictive values were calculated if a significant cut-off value was detected with a 95% confidence interval and a 5% level of significance.

## Results

A total of 301 participants are included in the study with a dominance of females (63.8% vs 36.2%). Seventy-four subjects (24.6%) were frail and the prevalence of frailty in men and women was 13.8% and 30.7%, respectively. The demographic and clinical characteristics of the participants according to frailty status are presented in Table 1.

**Table 1:** Sociodemographic and clinical characteristics of participants according to the frailty status

Sample characteristics (*n* = 301)	Robust (*n* = 102)	Pre-frail (*n* = 125)	Frail (*n* = 74)	*p* value
*Gender, n (%)*				
Female	47 (46.1)^a^	86 (68.8)^b^	59 (79.7)^b^	<**0.001**
Male	55 (53.9)^a^	39 (31.2)^b^	15 (20.3)^b^	
Age (years), median (min–max)]	70.5 (65–92)^a^	71 (65–88)^a^	76 (65–91)^b^	<**0.001**
*Education level, n (%)*				
5 years and less	73 (71.6)^a^	96 (76.8)^a,b^	65 (87.8)^b^	**0.036**
More than 5 years	29 (28.4)^a^	29 (23.2)^a,b^	9 (12.2)^b^	
*Education-level subgroups*				
Illiterate	12 (11.8)^a^	34 (27.2)^b^	24 (32.4)^b^	**0.004**
Literate (less than 5 years)	5 (4.9)^a^	11 (8.8)^a^	9 (12.2)^a^	
Primary school	56 (54.9)^a^	51 (40.8)^a^	32 (43.2)^a^	
Secondary school	10 (9.8)^a^	8 (6.4)^a^	6 (8.1)^a^	
High school	9 (8.8)^a,b^	15 (12)^b^	1 (1.4)^a^	
Undergraduate and more	10 (9.8)^a^	6 (4.8)^a^	2 (2.7)^a^	
*Marital status, n (%)*				
Married	71 (69.6)	73 (58.9)	39 (52.7)	0.062
Unmarried/widowed/divorced	31 (30.4)	51 (41.1)	35 (47.3)	
Smoking, *n* (%)	25 (24.5)	33 (26.4)	10 (13.5)	0.094
*Chronic diseases, n (%)*				
Hypertension	66 (64.7)^a^	91 (72.8)^a,b^	63 (85.1)^b^	**0.011**
Diabetes mellitus	33 (32.4)^a^	45 (36)^a^	45 (60.8)^b^	<**0.001**
Chronic pulmonary diseases	10 (9.8)^a^	16 (12.8)^a^	21 (28.4)^b^	**0.002**
Ischemic heart diseases	25 (24.5)	35 (28)	21 (28.4)	0.796
Congestive heart failure	0 (0.0)^a^	8 (6.4)^b^	12 (16.2)^b^	<**0.001**
Cerebrovascular diseases	0 (0.0)^a^	6 (4.8)^a,b^	6 (8.1)^b^	**0.008**
Malignancy	5 (4.9)	2 (1.6)	3 (4.1)	0.347
Dementia	4 (3.9)	3 (2.4)	5 (6.8)	0.288
*Anthropometric parameters [median(min–max)]*				
BMI (kg/m^2^)	27.43 (20.10–42.60)^a^	28.2 (16.50–48.70)^a,b^	30.25 (18.97–49.98)^b^	**0.01**
Calf circumference (cm)	36 (16–48)	35 (14–52)	36 (18–50)	0.355
*Daily activities [median(min–max)]*				
Katz ADL score	18 (8–18)^a^	18 (6–18)^a^	18 (6–18)^b^	<**0.001**
Lawton IADL score	8 (0–8)^a^	8 (0–8)^a^	7 (0–8)^b^	<**0.001**
*Nutritional status, n (%)*				
Normal nutrition	85 (83.3)^a^	78 (62.4)^b^	39 (52.7)^b^	<**0.001**
At risk of malnutrition	14 (13.7)^a^	42 (33.6)^b^	26 (35.1)^b^	
Malnutrition	3 (2.9)^a^	5 (4)^a,b^	9 (12.2)^b^	
*Physical functions, [median(min–max)]*				
Hand-grip strength (kg)	24.1 (8.9–41.1)^a^	20.0 (8.30–46.0)^b^	15.05 (5–38)^c^	<**0.001**
Usual gait speed (m/s)	1.0 (0.0–2.0)^a^	0.8 (0.17–2.0)^b^	0.57 (0–2)^c^	<**0.001**
*Physical activity, n (%)*				
Inactive	34 (33.3)^a^	66 (52.8)^b^	66 (89.2)^c^	<**0.001**
Minimally active	34 (33.3)^a^	38 (30.4)^a^	7 (9.5)^b^	
Active	34 (33.3)^a^	21 (16.8)^b^	1 (1.4)^c^	
*Biochemical parameters [median(min–max)]*				
Hemoglobin (g/dl)	14.0 (10.1–19.3)^a^	13.6 (8.1–17.7)^b^	12.5 (7.5–16.3)^c^	<**0.001**
Creatinine clearance (ml/min/1.73 m^2^)	79.0 (23.0–102.0)^a^	78.0 (33.0–102.0)^a,b^	71.5 (16–97)^b^	**0.011**
Albumin (mg/dl)	45.0 (29.0–52.0)^a^	45.0 (33.4–62.9)^a^	43.1 (28.2–52.8)^b^	**0.002**
CRP (mg/l)	2.55 (0.30–66.0)^a^	2.70 (0.20–104.0)^a,b^	3.6 (0.4–362)^b^	**0.036**

Bolds are statistically significant values

*BMI* body mass index; *ADL* activity of daily living; *IADL* instrumental activity of daily living; *CRP* C-reactive protein

^a,b,c^ The groups with the same letters within a column are not significantly different according to pairwise comparisons

The results of ultrasonographic measurements of RFM among robust, pre-frail, and frail groups were summarized in Table 2. Frail and pre-frail participants had significantly lower thickness (*p* = 0.002), CSA (*p* = 0.009), and fascicle length (*p* = 0.043) compared to robust participants. PA was significantly different among the 3 groups (*p* < 0.001), lowest in the frail group.

**Table 2:** Ultrasonographic measurements of rectus femoris among robust, pre-frail, and frail groups

Ultrasonographic findings of RF (mean ± SD) (*n* = 301)	Robust (*n* = 102)	Pre-frail (*n* = 125)	Frail (*n* = 74)	*p* value
Thickness (mm)*	12.45 ± 2.58^a^	11.6 ± 2.48^b^	11.12 ± 2.32^b^	**0.002**
CSA (mm^2^)**	4.2 (1.7–10.8)^a^	3.9 (1.6–7.9)^a,b^	3.7 (1.8–7.2)^b^	**0.009**
Muscle stiffness (kPa)**	15.25 (3.63–33.0)	15.75 (5.15–30.5)	14.38 (4.25–35.25)	0.802
Fascicle length (mm)**	55.0 (39.0–70.0)^a^	53.0 (38.0–66.0)^b^	53.7 (36.3–66.7)^a,b^	**0.043**
Pennation angle (degree)*	11.19 ± 2.12^a^	10.12 ± 2.27^b^	9.14 ± 1.85^c^	<**0.001**
Hyperechogenicity (*n*/%)	8 (7.8)	9 (7.2)	10 (13.5)	0.285

Bolds are statistically significant values

*RF* rectus femoris muscle; *CSA* cross-sectional area

^*^Mean ± SD

^**^ Median(min–max)

^a,b,c^ The groups with the same letters are not significantly different according to pairwise comparisons

There was a significant positive correlation between the FRAIL questionnaire’s score and age, BMI, and CRP (*r* = 0.282, *p* < 0.001; *r* = 0.148, *p* = 0.010; *r* = 0.135, *p* = 0.019, respectively). Katz/Lawton scores, hand-grip strength, usual gait speed, creatinine clearance, level of hemoglobin, and albumin were negatively correlated with the FRAIL score. In terms of ultrasonographic assessments, the thickness, CSA, and fascicle length of RFM were negatively correlated with FRAIL score (*r* = −0.216, *p* < 0.001; *r* = −0.198, *p* < 0.001; and *r* = −0.129, *p* = 0.025, respectively). PA also had a negative but a weak correlation with the FRAIL score (*r* = −0.358, *p* < 0.001). Other ultrasonographic measurements were not correlated with the FRAIL score (*p* > 0.05).

Predictive factors for frailty identified by univariate and multivariable logistic regression analysis were reported in Table 3. The multivariable logistic regression analysis showed that, BMI (OR = 1.06, 95% CI: 1.01–1.13, *p* = 0.028), hemoglobin level (OR = 0.77, 95% CI: 0.64–0.94, *p* = 0.010), and PA (OR = 0.83, 95% Cl: 0.70–0.97, *p* = 0.019) were independent predictors of frailty.

**Table 3:** Predictive factors for frailty identified by univariate and multivariable logistic regression analysis

	Univariable logistic regression analysis			Multivariable logistic regression analysis		
	Crude OR	95% CI	*p* value	Adjusted OR	95% CI	*p* value
Age (years)	1.107	1.06–1.16	<**0.001**	–	–	–
Male sex	2.780	1.49–5.20	**0.001**	–	–	–
BMI (kg/m^2^)	1.068	1.02–1.12	**0.008**	**1.066**	1.007–1.129	**0.028**
MNA-SF score	0.824	0.74–0.92	**0.001**			
Hand-grip strength (kg)	0.071	0.03–0.19	<**0.001**	–	–	–
Usual gait speed (m/sec)	1.854	1.59–2.17	<**0.001**	–	–	–
CRP (mg/l)	1.010	1.00–1.02	0.137	–	–	–
Hemoglobin (g/dl)	0.685	0.58–0.81	<**0.001**	**0.772**	0.635–0.939	**0.010**
Thickness of RF (mm)	0.868	0.78–0.97	**0.011**	–	–	–
CSA of RF (mm^2^)	0.785	0.64–0.97	**0.025**	–	–	–
Muscle stiffness (kPa)	0.999	0.96–1.04	0.968			
Fascicle length (mm)	0.973	0.93–1.01	0.186	–	–	–
Pennation angle (degree)	0.724	0.63–0.83	<**0.001**	**0.826**	0.703–0.970	**0.019**
Echogenicity	1.930	0.84–4.43	0.120	–	–	–

Bolds are statistically significant values

BMI: Body mass index, MNA-SF: Mini nutritional assessment short form, CRP:C-reactive protein, RF: Rectus femoris muscle, CSA: Cross sectional area

**Fig. 1:**
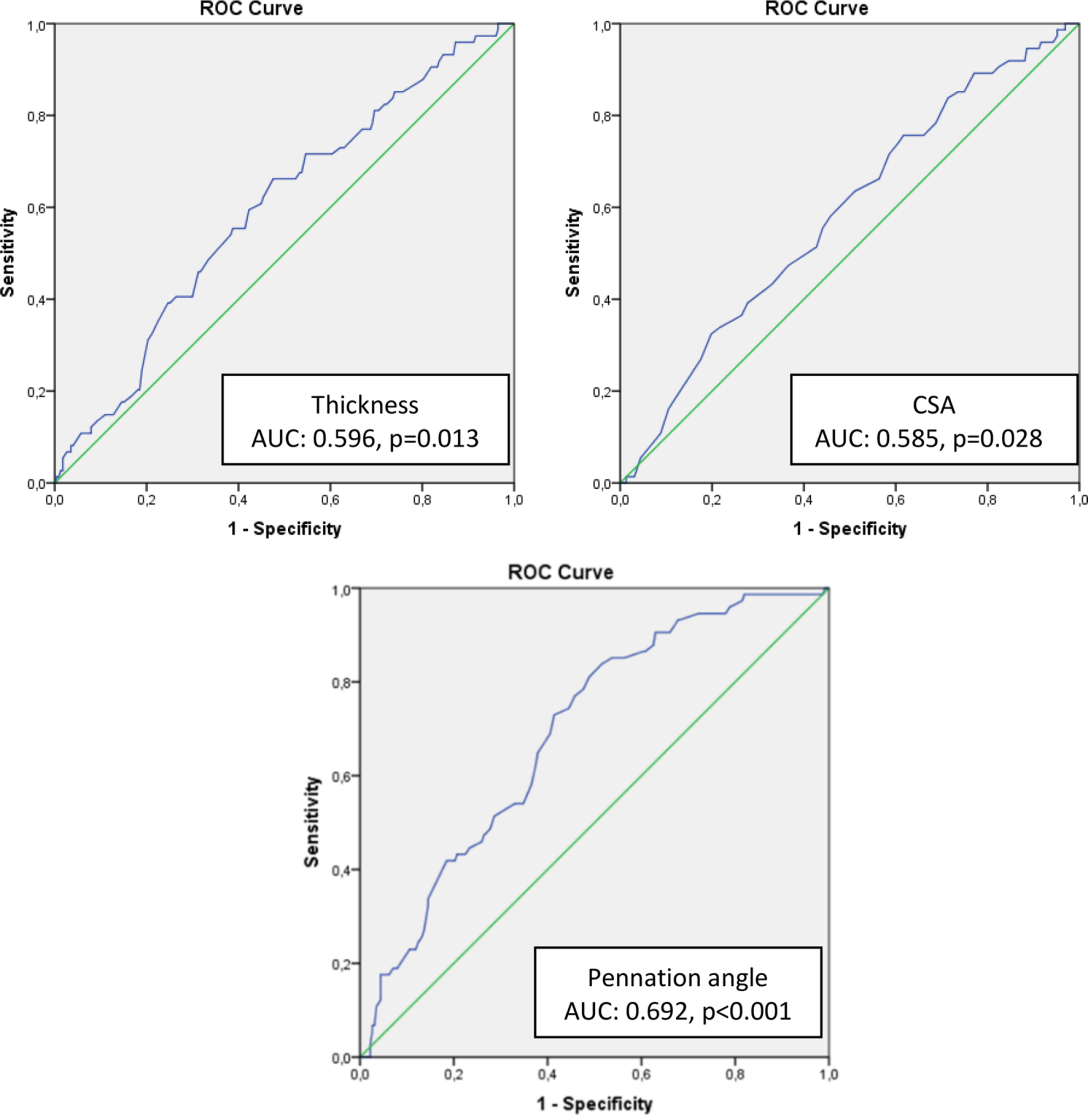
Receiver operating characteristic (ROC) curves presenting the ability of the thickness, CSA, and pennation angle of RFM to predict frailty

ROC analysis was performed on the results of ultrasonographic measurements of RFM (thickness, CSA, fascicle length, and PA) to interpret frailty (Table 4). Results of ROC analysis demonstrated a satisfactory result between the PA and frailty (AUC = 0.692, *p* < 0.001) (Fig. 1). The optimal cut-off values of RFM thickness, CSA, and PA to predict frailty was 11.75 mm, 4.55 mm^2^, and 10.65 degrees, respectively with 83.8% sensitivity and 48.4% specificity.

**Table 4:** ROC analysis of the ultrasound parameters in predicting frailty

	AUC	Sensitivity	Specificity	PPV	NPV
Thickness (mm)	0.596	66.22	52.42	31.21	82.64
CSA (mm^2^)	0.585	75.68	38.33	28.57	82.86
Pennation angle (degree)	0.692	83.78	48.46	34.64	90.16

*CSA* cross-sectional area; *AUC* area under curve; *PPV* positive predictive value; *NPV* negative predictive value

## Discussion

The main finding of the current study is the identification of a possible novel, non-invasive, ultrasound-based marker of frailty. Ultrasonographic measurement of the PA of RFM in community-dwelling older adults may predict frailty, as lower PA degrees were found to be related with frailty in older adults. Besides, according to our knowledge, it was the first study that investigated the relationship between the frailty and ultrasonographic parameters of RFM, in terms of not only thickness and cross-sectional area but also other parameters such as fascicle length, muscle stiffness, and PA.

It is known that aging is related to not only muscle loss but also critical changes in muscle structure [25]. RFM was evaluated commonly in previous studies to measure muscle mass or diagnose sarcopenia [10, 26]. However, the relation between ultrasonographic measurements of RFM and frailty was rarely investigated [27]. In the current study, muscle thickness, CSA, fascicle length, and PA, but not muscle stiffness, were found to be significantly lower in frail subjects compared to non-frail ones. In the previous studies, muscle thicknesses of thigh muscles were found to be related to frailty, as frail subjects had lower muscle thickness compared to normal and pre-frail individuals, which was consistent with our results [13, 15]. On the other hand, apart from the current study, these studies evaluated more than one thigh muscles but measured only the thickness of muscles. A recent study with a limited number of participants showed no significant difference in thickness between frail and non-frail groups but found an increased structural asymmetry in frail people [14]. These results may indicate that, especially in frail older people, evaluation of muscle from one aspect may not be enough to assess the structural changes in the muscle. Therefore, different parameters indicating muscle quantity and quality were evaluated in the current study.

PA was found to be lower in frail older individuals and reported to be a possible predictive ultrasonographic marker for frailty according to the results of the current study. According to our present knowledge, it was the first study that investigated the relationship between PA and frailty status in older people. PA and fascicle length are found to be a reliable parameter of muscle architecture in recent years [28]. PA may determine the maximum force of muscle contraction and supplies information about muscle quality and functional capacity. Smaller PA values are related to a lower generation capacity of strength [29, 30]. PA has been investigated in sarcopenia studies in recent years and showed a significant positive correlation with both muscle performance and strength parameters. Apart from muscle thickness and cross-sectional area, PA may be seen as a crucial parameter because it is closely related with muscle architecture [31]. In terms of fascicle length, no significant relationship was found in the current study.

Our results pointed out that lower PA of RFM (cut-off: 10.65 degrees) derived from ultrasound, may be used as a screening test for frailty with moderate-high sensitivity. The current methods to diagnose frailty are either subjective (depending on the patient’s declaration as in FRAIL), time-consuming (as in frailty index), or depend on patients’ cross-sectional performance [4, 32]. Although we already have multiple methods, the clinicians will have the opportunity to assess frailty objectively and apart from the unique performance of the patient which may be affected by the current health status, acute diseases, fluid balance or test cooperation, by this alternative ultrasonographic method [9]. These results may indicate that like sarcopenia, frailty may be evaluated in terms of objective muscle measurements. Further studies assessing the structural muscle changes in pre-frail and frail older people may assist early diagnosis of frailty and management of early interventions for the future.

PA may be affected by increased fat deposition especially in obese individuals and it was reported to be higher in obese older adults than normal-weight or underweight individuals [33]. In the current study, although having a significantly higher BMI, the frail group had a lower pennate angle compared to non-frails, which was statistically significant. Achieving this significance despite the higher BMI in the frail group, made the current results more reliable. PA is reported to have a higher reproducibility if it is measured by a trained radiologist and with a standardized protocol with the patient at rest and muscles relaxed [34]. In the current study, the protocol was standardized, and all measurements were done by the same trained researcher under the same physical conditions to minimize the risk of error. However, a commitment of an experienced and trained professional for performing an ultrasound may limit its importance/influence for being a screening test. There are very recent and promising studies about developing automatic measurement program for the pennation angle. In the future, ultrasonographic assessments may be used as a reliable and objective method more commonly by these programs [35].

Lower hemoglobin levels and higher BMI were also found to be independent predictors of frailty. Consistent with our findings, in a recent meta-analysis, being underweight and obese (BMI higher than 30) was found to be related to increased frailty risk [36]. Anemia and low hemoglobin levels are also well-known predictive factors for frailty in older adults [37, 38].

Echogenicity, which is thought to be a marker of muscle quality, is shown to be negatively correlated with muscle mass, hand-grip strength, and frailty in previous studies [15, 39]. The current study did not find any difference in the echogenicity of muscle among groups. This result may be related to the method that was used. In this study, radiologist reported whether there was an increase in echogenicity or not in the detected images, but in some studies echogenicity was measured using a computer-assisted grayscale analysis and reported quantitatively [15, 40]. Also, increased fatty infiltration of skeletal muscles in elderly people may cause misinterpretation in the assessment of echogenicity [41].

The current study had some limitations. First, high BMI in the study population may cause some misinterpretations such as higher measurements of thickness, surface area, and PA during the ultrasonographic assessments of RFM. This may have prevented us to report stronger relationships between ultrasonographic parameters and frailty. Second, as we know screening methods should be implemented quickly and practically by clinicians even in outpatient conditions, the need for an experienced sonographer and standardized protocol may complicate the use of the PA as a screening method. On the other hand, PA was shown to be sensitive diagnose frailty in community-dwelling older adults. Third, although the FRAIL and the ultrasound parameters were found to be negatively correlated, the relations were weak and further studies with larger samples are needed to confirm this correlation.

## Conclusion and implications

In conclusion, the results of the current study indicate that frailty was found to be related to changes in the skeletal muscle structure of the lower extremity. In the ultrasonographic assessment of RFM thickness, CSA, fascicle length and PA were significantly lower in frail individuals. Among the mentioned ultrasonographic parameters, only PA was found to predict frailty with moderate-high sensitivity. The current study is the first study that assessed the relationship between the frailty status and multiple ultrasound parameters of RFM at the same time. As a non-invasive, easily applicable, practical, and accurate method, ultrasound may be used to assess frailty in older people in the future.

## Data Availability

Our data are available if needed.
